# The complete mitochondrial genome of *Semilabeo notabilis* (Cyprinidae: Labeoninae) and phylogenetic implications

**DOI:** 10.1080/23802359.2018.1467231

**Published:** 2018-05-11

**Authors:** Lin Wu, Shengping Zhong, Hongyu Tan, Kaicheng Wang, Guangping Cheng, Xiuli Chen, Man Zhang

**Affiliations:** a Guangxi Colleges and Universities Key Laboratory of Aquatic Healthy Breeding and Nutrition Regulation, College of Animal Science and Technology, Guangxi University, Nanning, Guangxi, China;; b Key Laboratory of Marine Biotechnology, Guangxi Institute of Oceanology, Beihai, Guangxi, China;; c Guangxi Academy of Fishery Sciences, Nanning, Guangxi, China

**Keywords:** *Semilabeo notabilis*, mitogenome, phylogeny

## Abstract

*Semilabeo notabilis* is an Endangered cyprinid fish species in China. In this study, the complete mitochondrial genome of *S. notabilis* was firstly determined and described. It was 16,599 bp in length and composed by 13 protein-coding genes, 22 *tRNA* genes, 2 *rRNA* genes, and a control region (D-loop). The overall nucleotide composition was 31.9% of A, 15.7% of G, 25.4% of T, and 27.0% of C, with a total of 57.3% A + T content. Phylogenetic analysis suggested that *S. notabilis* had the closest evolutionary relationship with *S. obscurus*. The availability of the *S. notabilis* mitogenome would contribute to further molecular systematics and conservation genetics studies of this species.

*Semilabeo notabilis* is a valuable indigenous species of cyprinid fish that inhabit the gravel-bed rivers in southern China (Chen et al. 2000). Since 2012, *S. notabilis* has been listed on The IUCN Red List as the Data Deficient species (Huckstorf 2012), and then evaluated as an Endangered species in the latest red list of China’s vertebrates (Jiang et al. 2016). Till date, the available genetic information of *S. notabilis* is limited and no complete mitogenome has been reported. In this study, we provided the first characterization of the complete mitochondrial genome of *S. notabilis*, contributing to phylogenetics and conservation biology researches of this fish species.

The sample of *S. notabilis* was collected from Bama Yao Autonomous County (24°08′33.64″N, 107°15′34.74″E) in the upper drainage basin of the Pearl River (Guangxi, China). Muscle specimens were fixed in 95% ethanol and preserved in Guangxi Colleges and Universities Key Laboratory of Aquatic Healthy Breeding and Nutrition Regulation, Guangxi University. Total genomic DNA was extracted through Genomic DNA Isolation Kit (QIAGEN, Hilden, Germany). Ten pairs of primers were designed to amplify the PCR products for sequencing. Mitochondrial genome was assembled by Sequencher software (Gene Codes, Ann Arbor, MI, USA), and then annotated using MitoAnnotator (Iwasaki et al. 2013).

The complete mitogenome of *S. notabilis* was 16,599 bp in length (GenBank accession no. MH048527). Genomic organization showed high degree of conservation among *S. notabilis* and other Labeoninae fishes, including 13 protein-coding genes (PCGs), 22 *tRNA* genes, 2 *rRNA* genes, and a A + T-rich control region (D-loop). The whole mitogenome consisted of 31.9% A, 15.7% G, 25.4% T, and 27.0% C, with a slight AT bias (57.3%). As in many Labeoninae fish mitogenomes, the typical ATG initiation codon appeared in all the coding genes except in *COI* gene, having GTG as the start codon. Six PCGs had the common TAA termination codon and *ND6* gene had the complete TAG stop codon, whereas the remaining six were terminated by incomplete stop codon T or TA. All tRNAs showed the typical clover-leaf structure except for *tRNA^Ser(AGY)^* lacking the dihydrouridine (DHU) stem. The *12S* and *16S rRNA* were located between *tRNA^Phe^* and *tRNA^Leu (UUR)^*, and spaced by *tRNA^Val^*, with a size of 956 and 1686 bp, respectively. The D-loop was located between *tRNA^Pro^* and *tRNA^Phe^* with 937 bp long and 66.7% A + T content.

To validate the evolutionary position of *S. notabilis*, a phylogenetic tree inferred from 13 mitochondrial PCGs of 22 Labeoninae species was constructed by MEGA 7.0 with maximum-likelihood method (Kumar et al. 2016). Two species of Barbinae were included as out-groups. Evolutionary model selection was inferred using PhyML 3.0, where the substitution model GTR + G + I was resulted as the best-fit model (Guindon et al. 2010). Phylogenetic analysis showed that *S. notabilis* had a sister relationship with *S. obscurus* (Figure 1), which was in accordance with the traditional morphological classification and recent molecular works (Zhou and Zhang 2006; Yang et al. 2012). The mitogenomic resource obtained in the present study will provide preliminary molecular data for further genetic investigations and planning effective conservation strategies of *S. notabilis*.

**Figure 1.**
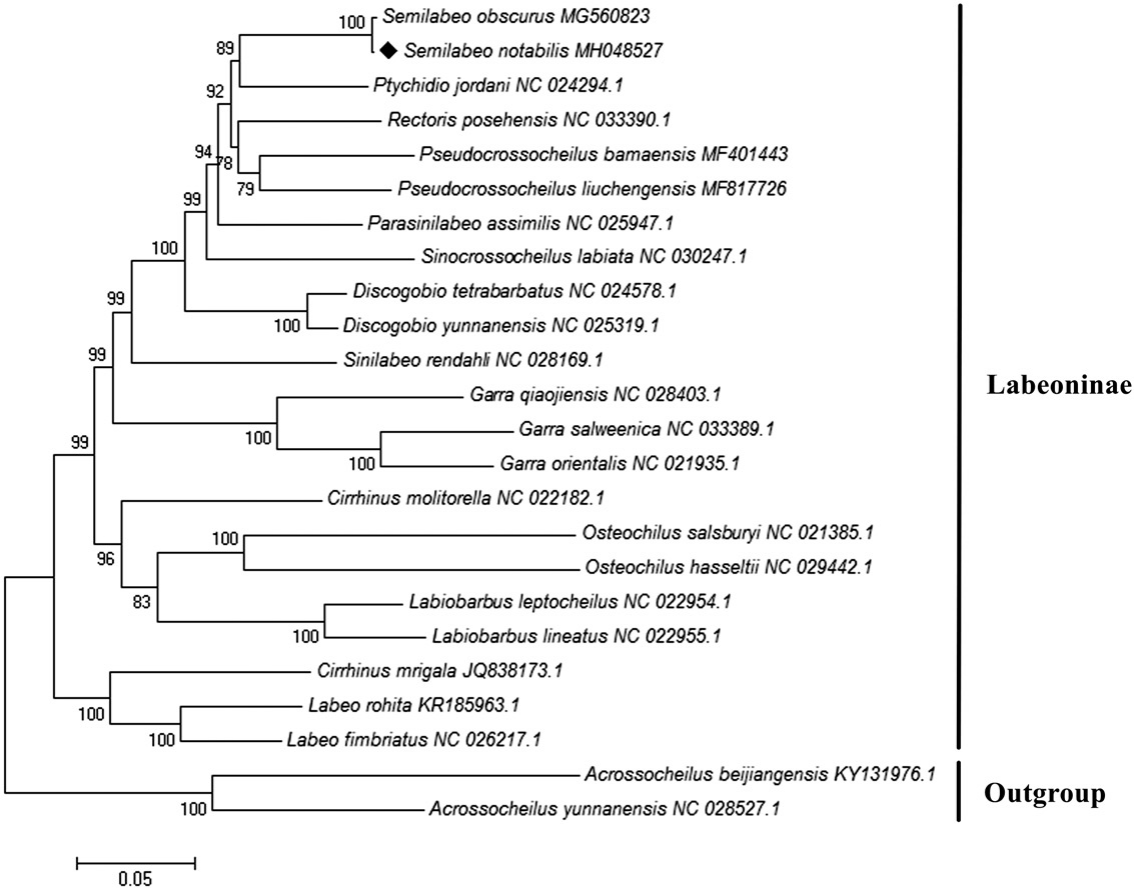
Maximum-likelihood tree of the phylogenetic relationships among 22 Labeoninae species based on 13 concatenated PCGs with GTR + G + I model. Two species of Barbinae were included as out-groups. The number on branches indicates posterior probabilities in percentage. The number after the species name is the GenBank accession number. The mitogenomic information of *Semilabeo notabilis* is marked with rhombus.
